# The impact of the COVID-19 pandemic on patients with juvenile idiopathic inflammatory myopathies

**DOI:** 10.1186/s12969-023-00873-0

**Published:** 2023-09-12

**Authors:** Dawn M. Wahezi, Dominique Jerome, Evin Rothschild, Belina Yi, Jeffrey Dvergsten, Stacey Tarvin, Susan Kim, Tamar Rubinstein, for the CARRA JDM workgroup

**Affiliations:** 1 Division of Pediatric Rheumatology, Children’s Hospital at Montefiore, 3415 Bainbridge Avenue, Bronx, NY USA; 2 Children’s Hospital Los Angeles, Los Angeles, CA USA; 3 Duke Children’s Health Center, Durham, NC USA; 4 Riley Children’s Health, Indianapolis, IN USA; 5 UCSF Benioff Children’s Hospital, San Francisco, CA USA

**Keywords:** Pediatric rheumatology, Juvenile idiopathic inflammatory myopathies

## Abstract

**Background:**

Throughout the COVID-19 pandemic, there have been concerns regarding the risks of infection in patients with autoimmune disease. In this study, we investigated the impact of the pandemic on patients with juvenile idiopathic inflammatory myopathies (JIIM).

**Methods:**

Data were collected using a patient/caregiver survey via Research Electronic Data Capture (REDCap) database. Eligibility included JIIM diagnosis and current age less than 21 years old. Surveys were distributed via the CureJM organization, social media, Childhood Arthritis and Rheumatology Research Alliance (CARRA) network and Dr. Peter Dent Pediatric Rheumatology Bulletin Board.

**Results:**

Eighty-four respondents accessed the survey, 70 (83%) consented to participate, and 54 out of 70 completed the full survey (77%). Twenty-seven out of 57 patients (47%) tested positive for COVID-19, with 7 (12%) testing positive more than once. Despite broad usage of immunosuppressive medications, 24 out of 27 (89%) reported mild symptoms with none requiring hospitalization. Four patients reported a flare of JIIM symptoms after COVID-19; three of whom held immunomodulatory medications during their infection. Thirty-seven out of 54 respondents (69%) reported vaccination against COVID-19, with 9 out of 37 (24%) reporting minor vaccine side effects and one reporting JIIM flare post vaccination. Twenty-one out of 54 (39%) respondents reported psychosocial concerns related to the COVID-19 pandemic.

**Conclusions:**

Patients with JIIM, including those on multiple immunosuppressive medications, had mild symptoms related to COVID-19. Most patients tolerated COVID-19 vaccination well. Few patients had disease flare post-COVID-19 or vaccination. Mental health concerns were demonstrated in JIIM patients during the COVID-19 pandemic.

**Supplementary Information:**

The online version contains supplementary material available at 10.1186/s12969-023-00873-0.

Since the onset of the coronavirus disease 2019 (COVID-19) pandemic, there have been concerns regarding the risks of infection with severe acute respiratory syndrome coronarvirus 2 (SARS-CoV-2) in patients with autoimmune conditions and particularly in those on immunosuppressive therapy. Numerous questions arose surrounding safe access to medical care, community risk reduction to prevent exposure to SARS-CoV-2, and modification of immunosuppressive therapy to decrease the risk of severe complications from COVID-19. Although data are mixed, reports in the literature of patients with adult-onset rheumatic disease suggest that patients with conditions such as systemic lupus erythematosus [1] as well as those on immunosuppressive therapies, such as rituximab, cyclophosphamide and mycophenolate mofetil, may be at higher risk of poor outcomes [2, 3]. To date, there is little evidence to suggest that children with rheumatic disease are at increased risk of more severe outcomes due to COVID-19.

In addition to concerns directly related to COVID-19, investigations in patients with idiopathic inflammatory myopathies (IIM) and juvenile idiopathic inflammatory myopathies (JIIM), have also explored the relationship of SARS-CoV-2 as a viral trigger implicated in the underlying pathogenesis of these conditions. Given the overlapping clinical manifestations, including cutaneous features, myositis, interstitial lung disease, and myocarditis, as well as activation of the interferon (IFN) pathway, SARS-CoV-2 has been speculated to be responsible for an increased incidence [4] and disease flare in patients with IIM/JIIM [5, 6] throughout the COVID- 19 pandemic. Additional considerations for these observations include reports of patients expressing apprehension about safe access to the hospital system, medication access difficulties [7] and delays to clinical care, simultaneously contributing to the risk of exacerbating the underlying disease during the COVID-19 pandemic [8].

To date, there are few studies examining the overall impact of the COVID-19 pandemic in children and adolescents with JIIM with limited data on health outcomes related to COVID-19, the potential for disease flares, and the association with psychosocial morbidity in this population. Furthermore, there are fewer studies examining the COVID-19 pandemic from the perspective of patients with JIIM and their caregivers. In this study, we investigated the impact of the COVID-19 pandemic on children and adolescents with JIIM, specifically examining outcomes related to SARS-CoV-2 exposure, COVID-19, COVID-19 vaccination, as well as the impact on overall psychosocial well-being.

## Patients and methods

### Population

Participants were eligible if they were diagnosed with JIIM, under the age of 21 years old at the time of the survey and had history of SARS-CoV-2 exposure, COVID-19 and/or COVID-19 vaccination. Caregivers were required to complete surveys for participants under 18 years old. After obtaining institutional review board (IRB) approval, participants were recruited via patient distribution lists within the CureJM organization and via social media. Pediatric rheumatology providers were also recruited via the Childhood Arthritis and Rheumatology Research Alliance (CARRA) network and the Dr. Peter Dent Pediatric Rheumatology Bulletin Board to assist with survey distribution. All participants provided written consent to participate in the study.

### Patient/Caregiver survey

Data were collected using a patient/caregiver survey in English and Spanish via Research Electronic Data Capture (REDCap) database (supplemental table). Survey components included six topic domains: (1) Demographics: age, sex, race, ethnicity, and additional comorbidities that pose increased risk of severe outcomes related to COVID-19; (2) JIIM disease features: subtype, primary JIIM manifestations, myositis specific autoantibodies (MSA), medications prior to COVID-19 and patient global assessment scale (GAS) prior to COVID-19; (3) SARS-CoV-2 exposure: close contacts, medications modifications, and patient GAS after exposure; (4) COVID-19: testing, number of infections, symptoms, hospitalization data, medication modifications, and patient GAS after COVID-19, as well as data on disease flare after COVID-19; (5) COVID-19 vaccination: type of vaccine, side effects, disease flare after vaccine, medication modification, and patient GAS after vaccination; (6) Overall impact of the COVID-19 pandemic: access to medical care, as well as psychological and/or emotional impacts due to the COVID-19 pandemic. In the final question of the survey, participants were provided a free text option to include any “additional comments or concerns regarding COVID-19” to be used in thematic analysis.

### Statistical analysis

Statistical analysis was performed with STATA software, version 15.1. Descriptive statistics were performed, including median and interquartile range (IQR) for non-normally distributed continuous variables, and frequencies and percentages for categorical variables. Non-parametric testing was performed using the Wilcoxon signed-rank test to examine for changes in patient GAS. Chi-square and Fischer’s exact were used to assess comparisons using categorical variables. Thematic analysis [9] was used to examine themes across the qualitative data collected in the final question of the survey. Three researchers (DW, ER, TR) independently coded qualitative data to identify, analyze and interpret pattern responses and themes, followed by a discussion to resolve any discrepancies.

## Results

### Survey respondents

Eighty-four respondents accessed the survey, with 70 (83%) consenting to participate. Among those who consented, 54 completed the full survey (77%). Surveys were primarily completed in English (98%), by parents/caregivers (93%) and with the majority of patients reported as female sex (70%), white (74%) and non-Hispanic (64%) (Table 1).

**Table 1:** Demographics and JIIM baseline features (n = 70)*

	N (%)
**Sex**	
Female	49 (70)
Male	20 (29)
Prefer not to answer	1 (1)
**Current age of JIIM patient**	
Under 5	5 (7)
6–10 years	22 (31)
11–14 years	20 (30)
15–17 years	11 (16)
18–21 years	12 (17)
**Race (n = 68)**	
White	50 (74)
Black/African American	7 (10)
American Indian	1 (1)
Asian	2 (3)
Other Race	4 (6)
Prefer not to answer	4 (6)
**Ethnicity**	
Hispanic	22 (31)
Non-Hispanic	45 (64)
Prefer not to answer	3 (5)
**JIIM Subtype (n = 62)**	
JDM	59 (95)
JPM	3 (5)
**JIIM clinical manifestations at diagnosis (n = 62)**	
Skin Disease	52 (84)
Muscle Disease	54 (87)
Gastrointestinal Involvement	7 (11)
Lung Involvement	9 (15)
Heart Involvement	6 (10)
Joint Disease	16 (26)
**JIIM autoantibodies (n = 63)**	
P155/140 (TIF-1)	8 (13)
MJ (NXP-2)	5 (8)
Jo-1 (anti-synthetase)	2 (3)
Mi-2	1 (2)
MDA-5 (CADM-140)	4 (6)
Other	3 (5)
Negative Antibodies	3 (5)
Unknown or not done	37 (59)
**Medications during 6 months prior to COVID-19 exposure/infection (n = 62)**	
Steroids	25 (40)
Methotrexate	34 (55)
Hydroxychloroquine	26 (42)
Intravenous immunoglobulin (IVIG)	25 (40)
Mycophenolate mofetil	17 (27)
Rituximab	11 (18)
Anti-TNF agent	1 (2)
Calcineurin Inhibitors	2 (3)
Other**	6 (10)
None of the above	8 (13)
**Total number of immunosuppressive medications prior to exposure/infection with COVID-19 (n = 62)**	
None	9 (15)
One	3 (5)
Two	24 (39)
Three or more	26 (42)
*N = 70 participants, except where otherwise indicated. **Other medications include abatacept (n = 1), leflunomide (n = 1) and tofacitinib (n = 2).	

### JIIM history

The majority of patients (n = 70) were reported as having juvenile dermatomyositis (JDM) (95%). Median disease duration of JIIM was 3.97 years [IQR: 2.03, 7.96], with a range from 0.25 to 16.09 years. Primary JIIM manifestations, myositis specific autoantibodies and medications taken within 6 months prior to COVID-19 are listed in Table 1. Median number of baseline medications was 2 [IQR: 2, 3] with a maximum baseline of 5 medications, reported in 4 patients. A total of 81% of respondents reported being on two or more immunosuppressive medications prior to COVID-19.

### COVID-19 exposure

Among respondents, 44 out of 58 (76%) reported a known exposure to SARS-CoV-2 during the COVID-19 pandemic. The most common exposure (n = 44) was a household contact (52%), followed by a relative/friend (39%) and a school contact (32%). Among those who had a known SARS-CoV-2 exposure (n = 44), 24 (55%) report testing positive for COVID-19 themselves after the exposure.

### COVID-19

**Fig. 1:**
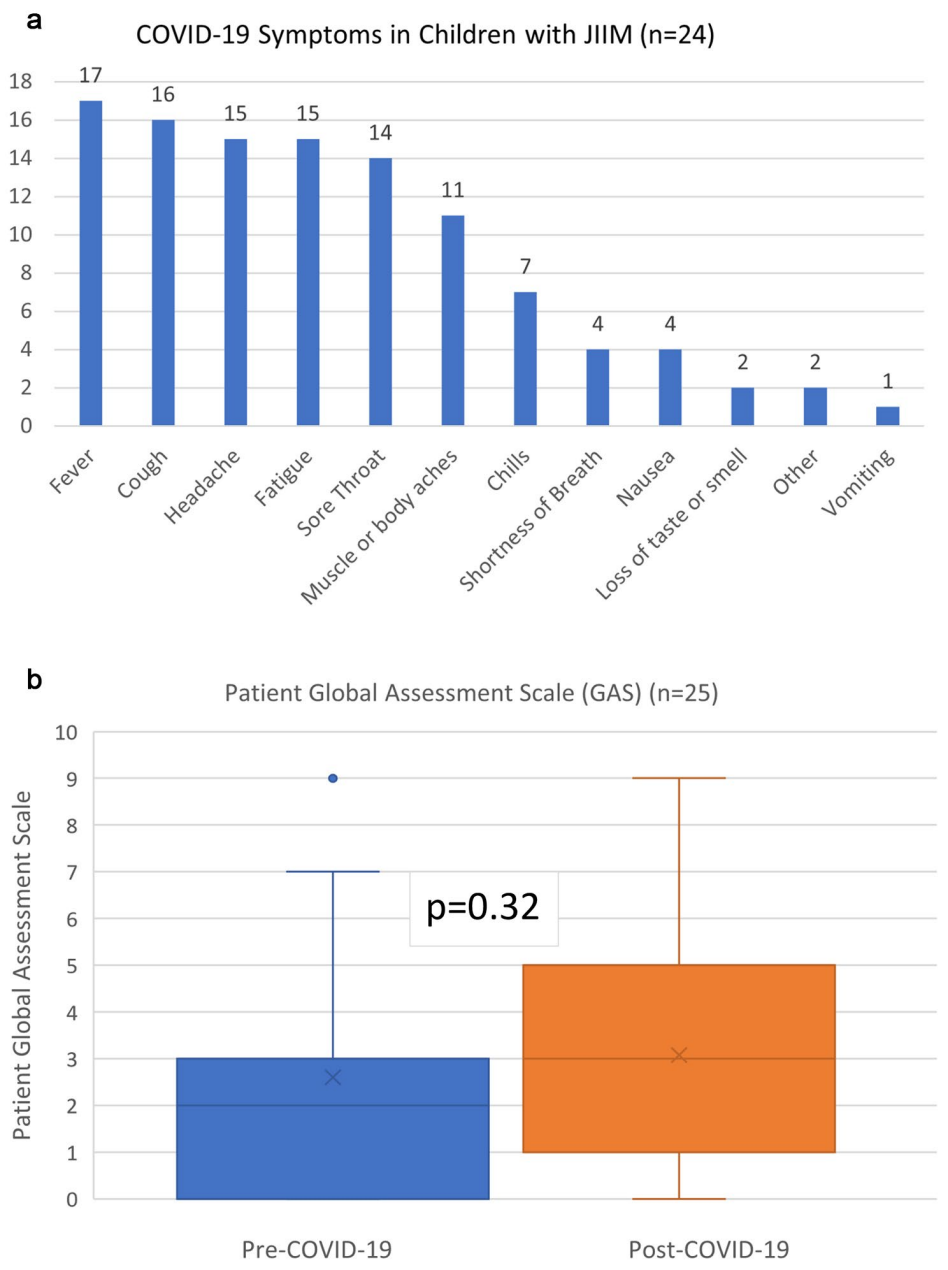
(**a**) Twenty-four patients out of 27 who tested positive for COVID-19 reported acute COVID-19 symptoms. Total 108 symptoms were reported (**b**) Global assessment scale (GAS) for JIIM disease activity reported by participants pre- and post-COVID-19 (n = 25)

Among respondents, 27 out of 57 (47%) reported testing positive for COVID-19, with 7 out of 27 (26%) testing positive on more than one occasion. The majority (89%) reported typical symptoms of fever, cough, headache and fatigue (Fig. 1a). No patients were hospitalized or received medications specifically to treat COVID-19. Four respondents (15%), all of whom were female, white, and non-Hispanic, reported a flare of JIIM symptoms after COVID-19 including rash, weakness, myalgias/arthralgias and abdominal pain; three of these respondents reported holding immunomodulatory medications for 7–14 days in the context of active COVID-19 (Table 2). Among patients who tested positive for COVID-19, there was minimal change in patient global assessment scale (GAS) prior to and post-COVID-19 (median GAS [IQR]: 2 [0,3] and 3 [1,5] respectively; p = 0.32) (Fig. 1b).

**Table 2:** Features of JDM patients who reported flare of underlying disease post COVID-19 (n = 4; all were female, white, and non-Hispanic)

Age group (years)	Disease duration (years)	MSA	Baseline medication	COVID vaccine	Patient GAS prior to COVID-19	Patient GAS post COVID-19	COVID-19 positive more than once	Meds held for COVID-19 (days)
6–10	7.96	MJ	MTX, HCQ, IVIG	no	2	8	Yes, Milder 2nd time	Yes (7 days)
11–14	2.05	Unknown	Steroids, MTX, HCQ, IVIG, MMF	no	3	6	Yes, Milder 2nd time	Yes (14 days)
18–21	9.9	p155/140	HCQ, IVIG, Orencia	yes	3	5	No	Yes (14 days)
19–21	1.51	MDA-5	None	yes	1	9	Yes, diagnosed after first episode	No

### COVID-19 vaccination

Thirty-seven out of 54 respondents (69%) reported vaccination against COVID-19, with 9 out of 37 (24%) reporting minor vaccine side effects including arm pain, headaches, fever and chills.

One respondent (3%) reported JIIM flare post vaccination including rash, weakness and myalgia; this respondent did not report delaying JIIM medications in the context of COVID-19 vaccination.

### Medication modifications

Medication modifications made in response to SARS-CoV-2 exposure (n = 44), COVID-19 (n = 27) or COVID-19 vaccination (n = 37) included medications being held or delayed for 7–14 days in 16%, 26%, 22% respectively.

### Overall impact of the COVID-19 pandemic

Sixteen out of 54 respondents (30%) reported concerns related to either delayed appointments (n = 10), difficulty obtaining medication (n = 4) or avoidance of hospital care due to risk of exposure (n = 4). In addition, 21 out of 54 (39%) respondents reported psychosocial concerns related to the COVID-19 pandemic including anxiety (n = 18), depression (n = 13), stress (n = 12), social withdrawal (n = 8), irritability (n = 4), anger (n = 2) and two respondents endorsed suicidal ideation.

Themes expressed in final free text comments of the survey most commonly included stressors related to protective measures (such as masking and excessive cleaning) (n = 4), medications (n = 2), social isolation (n = 3) and school closures (n = 2). Themes also included concerns related to JIIM flare (n = 3) and COVID-19 vaccination (n = 2), as well as overall reassurance that COVID-19 resulted in mild illness in the JIIM patient (n = 6). Two patients expressed frustration regarding a lack of empathy from others. Representative excerpt quotes include: “It was a stressful time to be on immunosuppressants”; “The isolation was the worst part”; “although she did eventually contract covid-19….it did not have any negative impacts on her health and the situation ultimately relieved a lot of stress for her and our family to know that she came through it fine”.

## Discussion

To our knowledge, this is the first study to examine the overall impact of the COVID-19 pandemic on children and adolescents with JIIM and one of few studies to examine the pandemic from the perspectives of patients with JIIM and their caregivers. Based on our findings, patients with JIIM, including those on multiple immunosuppressive medications, had mild symptoms related to COVID-19 with none requiring hospitalization. Furthermore, only four patients out of 27 (15%) who tested positive for COVID-19 reported flare of their underlying disease after the infection. Three of the four patients who flared had withheld immunomodulatory medications in the context of active COVID-19, which could have contributed to the disease flare.

Despite initial concerns that children with rheumatic disease and/or those receiving immunomodulatory medications would be at increased risk of severe outcomes related to COVID-19, there has been limited data to suggest increased risk of hospitalization or severe illness [10–12]. Literature from the COVID-19 Global Rheumatology Alliance (GRA) indicates that adult patients with certain diseases (e.g. systemic lupus erythematosus), additional comorbidities (e.g. lung disease) or those receiving certain immunomodulatory medications (e.g. rituximab, cyclophosphamide, mycophenolate mofetil) may be at higher risk for worse outcomes [1, 2], however these findings are not consistent across studies and have not been replicated in children. Our survey results help to provide additional reassurance to patients with JIIM and their families, demonstrating that no JIIM patients in this cohort reported severe outcomes from COVID-19 and none required hospitalization, including those on numerous immunosuppressive medications.

A few reports suggest an increased incidence of JIIM within the era of the COVID-19 pandemic [4, 13]. Additional studies suggest increased risk of JIIM flare after COVID-19 infection [5, 6]. In our study, 4 respondents (15%) reported flare of underlying JIIM, with one respondent reporting initial onset of JIIM, in the context of active COVID-19. Symptoms of JIIM flare included worsening rash, weakness, muscle/joint pain and one respondent reporting gastrointestinal involvement. Disease flares were more likely to be after first episode of COVID- 19 and more likely in those who were instructed to hold their immunomodulatory medications in the context of an active infection. Despite reassuring data suggesting that children and adolescents with rheumatic disease are not at increased risk of severe outcomes from COVID-19, conventional clinical practice has been for providers to withhold immunosuppressive medications in the context of active infection. Given that symptoms from active COVID-19 can vary widely and there is limited data regarding the impact of immunosuppressive medications on those symptoms, clinical guidance provided by the American College of Rheumatology (ACR) recommends that disease-modifying antirheumatic drugs be held in cases of confirmed symptomatic COVID-19 [14]. It is suggested that medications may be restarted 7–14 days after resolution of fever and respiratory symptoms. In our survey, 7 out of 27 (26%) of patients held their immunosuppressive medications in the context of active, symptomatic COVID-19, with three patients developing JIIM flare. Given our limited sample size, it is difficult to determine which factors may have led to JIIM flare, including immune activation from COVID-19 infection, delaying of immunosuppressive medications or other factors not identified in this study.

There have been few case reports that describe new onset JIIM or disease flare after COVID-19 vaccination [15]. No larger studies have been published to verify that people with autoimmune disease are at higher risk of adverse reactions from COVID-19 vaccination, and the ACR clinical guidance recommends that children with rheumatic disease receive COVID-19 vaccination in accordance with current US Food and Drug Administration (FDA), Center for Disease Control (CDC) and local recommendations [14, 16]. In our survey, 69% of respondents report receiving the COVID-19 vaccination, with reports of minor side effects. One respondent (3%) reported JIIM flare after the 2nd dose of the Pfizer-BioNTech COVID-19 vaccine, requiring intensification of immunomodulatory medications to treat disease flare.

Accumulating evidence suggests an increased emotional burden experienced by patients with chronic disease throughout the pandemic [7, 17, 18]. This burden has been compounded by limited access to medical care, fears related to underlying illness and risks related to COVID-19, as well as stressors related to quarantine and social distancing. In a prior study, Wilkinson et al. surveyed household members of families with JDM and found disruption of medical treatment in 40% of patients. Parents and caregivers expressed themes of stress, fear and anxiety throughout the course of the pandemic [17]. Similarly, in our survey, disruption in medical care was reported in 30% of respondents and mental health concerns were reported by almost 40%, with caregivers most commonly discussing themes of stress related to protective measures and social isolation, as well as fears related to JIIM flare.

Finally, the respondents for this survey were made up of predominantly white and non-Hispanic patients, with 10% of respondents reporting black race and 31% reporting Hispanic ethnicity. Given that patients in black and Hispanic populations may have more complications related to JDM [19, 20] and were disproportionately impacted by the COVID-19 pandemic with higher risk for poor physical and mental health outcomes [7, 21], our study may be inadvertently biased to those with less severe outcomes. Larger investigation into a more diverse patient population would be beneficial in further evaluating these results.

There are several limitations to our study. First, given the rarity of JIIM, we had a relatively small sample size with only 70 respondents consenting to participate and only 54 completing the full survey. Second, our survey may not be generalizable to all patients with JIIM as it is inherently subject to sampling bias and non-response bias. We attempted to reduce bias by recruiting through various channels, however participants who respond to surveys may differ from those who do not. Finally, due to regulatory concerns, parents/caregivers were required to complete the survey for any children under 18 years of age, resulting in only 7% completion by individual patients. It has been demonstrated that discordance exists between patient and parental responses on patient experience surveys [22], and parental input may have impacted responses to measures, including the patient GAS and psychosocial impact questions. It is uncertain how these responses might vary with patient self-reporting.

## Conclusions

Our findings suggest that children and adolescents with JIIM, including those on numerous immunosuppressive medications, may not be at increased risk of severe outcomes from COVID-19. Furthermore, very few patients experienced disease flare after COVID-19 or COVID-19 vaccination. Additional investigations are needed to examine the impact of COVID- 19 on a more diverse population of children and adolescents with JIIM and to further investigate strategies to reduce the mental health burden on these children and their families.

### Electronic supplementary material

Below is the link to the electronic supplementary material.

Supplementary Material 1

## Data Availability

The data generated in this study is not publicly available due to individual privacy, but portions of the data may be made available from the corresponding author upon reasonable request.

## List of abbreviations

ACR: American College of Rheumatology
CARRA: Childhood Arthritis and Rheumatology Research Alliance
CDC: Centers for Disease Control
COVID-19: Coronavirus disease 2019
FDA: Food and Drug Administration
GAS: Global assessment scale
GRA: Global Rheumatology Alliance
IFN: Interferon
IIM: Idiopathic inflammatory myopathies
IQR: Interquartile range
IRB: Institutional Review Board
JIIM: Juvenile idiopathic inflammatory myopathies
REDCap: Research Electronic Data Capture
SARS-CoV-2: Severe acute respiratory syndrome coronarvirus 2
