# Supplementary material for: The impact of the COVID-19 pandemic on patients with juvenile idiopathic inflammatory myopathies
Source: Pediatr Rheumatol Online J. 2023 Sep 12;21:100. doi: 10.1186/s12969-023-00873-0 (PMC10496159; doi:10.1186/s12969-023-00873-0)
Supplement: Supplementary file 1 — Supplementary Material 1 [file 12969_2023_873_MOESM1_ESM.pdf]

JIIM Covid Registry

Before completing this survey, please confirm the following:

1. The survey is being completed for a person with Juvenile Idiopathic Inflammatory Myositis (JIIM) (such as juvenile dermatomyositis, juvenile polymyositis or overlap myositis).

2. You are a person with JIIM under 21 years old OR the parent of a child with JIIM under 21 years old. Please note, this survey is intended to be completed by individuals 18+ years of age. If you are a minor (under 18 years old) and wish to continue, parental or guardian supervision is required.

AND

3. The person with JIIM has had at least ONE of the following
a. Close exposure to COVID-19
b. Infection with COVID-19
c. Completed COVID-19 vaccination

By clicking YES, you are affirming that all of these statements are true and you are eligible to complete the survey.

*This survey should take approximately 15 minutes to complete.

Please download and read consent

[Attachment: "Electronic_consent (1).pdf"]

---

1) Do you consent to participate?

○ Yes
○ No

Demographics

Please complete the survey below.

Thank you!

Who is filling out this survey? (patient must be under 21 years old)

◯ Patient (if over 18 years old)
◯ Parent/guardian

What is the patient's sex?

◯ Male
◯ Female
◯ Prefer not to answer

What age group does the patient fall in?

◯ Under 5 years old
◯ 6-10 years
◯ 11-14 years
◯ 15-17 years
◯ 18-21 years

What is the patient's race?

☐ American Indian
☐ Asian
☐ Black/African American
☐ Native Hawaiian or Other Pacific Islander
☐ White
☐ Other race
☐ Prefer not to answer

Other race (please specify)

____

What is the patient's ethnicity?

◯ Hispanic
◯ Non-Hispanic
◯ Prefer not to answer

Does the patient have any of the following conditions? Please check all that apply.

- ☐ Asthma
- ☐ Cancer
- ☐ Diabetes
- ☐ Sickle Cell Disease
- ☐ Lung disease
- ☐ Down Syndrome
- ☐ Heart Condition
- ☐ Neurologic Disorder
- ☐ Immunosuppression (due to medical condition or medications)
- ☐ Inherited Metabolic Disorders
- ☐ Obesity
- ☐ None of the above

JIIM Data

Please complete the survey below.

Thank you!

What was the approximate date of JIIM diagnosis? (If exact date is unknown, please estimate Month and Day)

____
(M-D-Y)

What subtype of JIIM?

◯ Juvenile dermatomyositis (JDM)
◯ Juvenile polymyositis (JPM)
◯ Overlap myositis
◯ Other, please specify_________________

Other, please specify________

____

What are the main symptoms of JIIM (please check all that apply):

☐ Skin disease
☐ Muscle disease
☐ Gastrointestinal involvement
☐ Lung involvement
☐ Heart involvement
☐ Joint disease
☐ None of the above

Has the patient ever tested postive for any of the following ? (Select all that apply)

☐ P155/140 (TIF-1)
☐ MJ (NXP-2)
☐ SRP
☐ Jo-1 (anti-synthetase)
☐ Mi-2
☐ MDA-5 (CADM-140)
☐ Other
☐ Negative Antibodies
☐ Unknown or not done

Which medications was the patient on PRIOR to COVID-19 exposure/infection (within the last 6 months prior to exposure/infection): Please check all that apply

☐ Steroids
☐ Methotrexate
☐ Hydroxychloroquine (plaquenil)
☐ IVIG
☐ Rituximab
☐ Mycophenolate mofetil (cellcept)
☐ Cyclophosphamide (cytoxan)
☐ Anti-TNF agent (etanercept (Enbrel), adalimumab (Humira), infliximab (Remicade)
☐ Calcineurin inhibitors (tacrolimus, sirolimus, cyclosporine)
☐ Other, please specify __________________
☐ None of the above

Other, please specify ______

____

Considering all the ways in which your JIIM affects you/your child, how would you rate the disease PRIOR to COVID-19 exposure/infection/vaccination:

None | Moderate | Severe

(Place a mark on the scale above)

Were there any recent medication changes PRIOR to COVID-19 exposure/infection:

◯ No, changes within 1 month prior to exposure/infection
◯ Yes, medications were increased within 1 month prior to exposure/infection
◯ Yes, medications were tapered within 1 month prior to exposure/infection
◯ Yes, medications were discontinued within 1 month prior to exposure/infection. If discontinued, please specify
____

Other, please specify ____

COVID exposure questions

Please complete the survey below.

Thank you!

Was the patient EVER in close contact with anyone who has tested positive for COVID-19 or who had symptoms of COVID-19?

◯ Yes
◯ No

Who was the contact for exposure(s)? (Check all that apply)

☐ Household member
☐ School
☐ Relative/Friend
☐ Other

Did the patient EVER test positive for COVID -19 AFTER exposure?

◯ Yes
◯ No
◯ Unsure

Which test was used?

◯ PCR (nasal swab, results up to 5 days)
◯ Rapid (nasal swab, results in 10-15 minutes)
◯ Antibody (blood test)
◯ Unknown or Not sure

Did your doctor advise you to modify your immunosuppressive medications due to exposure?

◯ Yes, medications were held or delayed
◯ Yes, medication doses were lowered
◯ Yes, medications were stopped
◯ No, medications were not changed

If medications were held, when were they restarted? After ____ days

____
(Enter number of days)

Considering all the ways in which your JIIM affects you/your child, how would you rate the disease AFTER COVID-19 exposure:

None Moderate Severe

(Place a mark on the scale above)

COVID positive questions

Please complete the survey below.

Thank you!

Did the patient test positive for COVID -19?

◯ Yes
◯ No

Did the patient test positive for COVID-19 more than once?

◯ Yes
◯ No

Please answer the following questions based on the time you were MOST symptomatic

Click NEXT to continue.

◯ NEXT

Which test was used?

◯ PCR (nasal swab, results up to 5 days)
◯ Rapid (nasal swab, results in 10-15 minutes)
◯ Antibody (blood test)
◯ Unknown or Not sure

Did the patient have any symptoms?

◯ Yes
◯ No

If yes, please check all that apply:

☐ Fever
☐ Headache
☐ Cough
☐ Sore throat
☐ Chills
☐ Shortness of Breath
☐ Fatigue
☐ Nausea
☐ Vomiting
☐ Loss of taste or smell
☐ Muscle or body aches
☐ Other (please specify)

If Other, please specify

Was the patient hospitalized for Covid-19 Infection?

◯ Yes
◯ No

If yes, How many days was the patient hospitalized?

____
(Enter number of days)

If yes, what were the total days of COVID-19 symptoms ?

____

During hospital stay, were there any complications from COVID-19?

◯ Yes
◯ No
◯ Unsure

If yes, please specify_____

Were any medications given specifically to treat COVID-19 (ie. remdesivir, steroids, monoclonal antibody, other)

◯ Yes
◯ No
◯ Unsure

If yes, please specify______

Did your doctor advise you to modify your immunosuppressive medications due to positive COVID testing?

◯ Yes, medications were held or delayed
◯ Yes, medication doses were lowered
◯ Yes, medications were stopped
◯ No, medications were not changed

If medications were held, when were they restarted? After______ days

____
(Enter number of days)

Did the patient have new or worsening JIIM symptoms after COVID-19 infection?

◯ Yes
◯ No

What were those symptoms? (Check all that apply)

☐ Rash
☐ Weakness
☐ Muscle/joint pain
☐ Abdominal pain
☐ Other

If other, please explain

If you tested positive more than once, describe a time when you were LESS symptomatic. (Date, symptoms, medication changes, etc)

Considering all the ways in which your JIIM affects you/your child, how would you rate the disease AFTER testing positive for COVID-19:

None | Moderate | Severe

(Place a mark on the scale above)

COVID vaccine

Please complete the survey below.

Thank you!

Did the patient receive the COVID-19 vaccine?

◯ Yes
◯ No

If yes, which vaccine product did they receive?

◯ Pfizer
◯ Moderna
◯ Janseen/Johnson & Johnson
◯ Other

Other, please specify___

____

If the vaccine received is a part of a two-dose series, did the patient receive BOTH doses?

◯ Yes, the patient received BOTH doses
◯ No, the patient only received first dose

When did the patient receive the last dose?

____
(M-D-Y)

Did the patient experience any side effects from the COVID-19 vaccine?

◯ Yes
◯ No

What side effects did the patient experience? Check all that apply.

☐ Pain
☐ Redness
☐ Swelling
☐ Headache/Fatigue
☐ Muscle Pain
☐ Chills
☐ Fever
☐ Nausea
☐ Other, please specify
☐ None of the above

Other, please specify

How soon after receiving the vaccine did the patient experience a reaction?

◯ Within 48 hours
◯ More than 48 hours but less than 1 week
◯ >1 week
◯ Not applicable

Did the patient have new or worsening JIIM symptoms after COVID-19 vaccination?

◯ Yes
◯ No

What were those symptoms? (Check all that apply)

☐ Rash
☐ Weakness
☐ Muscle/joint pain
☐ Abdominal pain
☐ Other

If Other, please explain

Did your doctor advise you to modify your immunosuppressive medications in relation to vaccine doses?

◯ Yes, medications were held or delayed
◯ Yes, medication doses were lowered
◯ Yes, medications were stopped
◯ No, medications were not changed

If medications were held, when were they restarted? After ____days

____
(Enter number of days)

Considering all the ways in which your JIIM affects you/your child, how would you rate the disease AFTER COVID-19 vaccination:

None — Moderate — Severe

(Place a mark on the scale above)

Did you/your child receive COVID-19 booster?

◯ Yes
◯ No
◯ Unsure

Impact of the pandemic overall

Please complete the survey below.

Thank you!

Was the patient impacted by any of the following? Please check all that apply.

☐ Delays to appointments
☐ Delays to lab assessments
☐ Difficulty obtaining medication
☐ Loss of insurance coverage
☐ Avoiding hospital care to risk exposure
☐ Other
☐ No impact

If other, please specify____

What point of access to healthcare did the patient utilize?

◯ Telehealth video
◯ In Person Visits
◯ Both
◯ Other

If other, please specify____

Did the patient experience any psychological or emotional impacts due to COVID-19 pandemic?

◯ Yes
◯ No

If yes, please check all that apply:

☐ Anxiety
☐ Depression
☐ Stress
☐ Suicidal Ideology
☐ Socially Withdrawn
☐ Irritability
☐ Excessive Anger

Are there any additional comments or concerns regarding Covid-19 you would like to share?
